# Integrating Bulk RNA and Single-Cell Sequencing Data Reveals Genes Related to Energy Metabolism and Efferocytosis in Lumbar Disc Herniation

**DOI:** 10.3390/biomedicines13071536

**Published:** 2025-06-24

**Authors:** Lianjun Yang, Jinxiang Li, Zhifei Cui, Lihua Huang, Tao Chen, Xiang Liu, Hai Lu

**Affiliations:** Department of Spine Surgery, The Fifth Affiliated Hospital, Sun Yat-sen University, Zhuhai 519000, Chinaeagle121@163.com (T.C.)

**Keywords:** lumbar disc herniation, energy metabolism, efferocytosis

## Abstract

**Background/Objectives**: Lumbar disc herniation (LDH) is the most common condition associated with low back pain, and it adversely impacts individuals’ health. The interplay between energy metabolism and apoptosis is critical, as the loss of viable cells in the intervertebral disc (IVD) can lead to a cascade of degenerative changes. Efferocytosis is a key biological process that maintains homeostasis by removing apoptotic cells, resolving inflammation, and promoting tissue repair. Therefore, enhancing mitochondrial energy metabolism and efferocytosis function in IVD cells holds great promise as a potential therapeutic approach for LDH. **Methods**: In this study, energy metabolism and efferocytosis-related differentially expressed genes (EMERDEGs) were identified from the transcriptomic datasets of LDH. Machine learning approaches were used to identify key genes. Functional enrichment analyses were performed to elucidate the biological roles of these genes. The functions of the hub genes were validated by RT-qPCR. The CIBERSORT algorithm was used to compare immune infiltration between LDH and Control groups. Additionally, we used single-cell RNA sequencing dataset to analyze cell-specific expression of the hub genes. **Results**: By using bioinformatics methods, we identified six EMERDEGs hub genes (IL6R, TNF, MAPK13, ELANE, PLAUR, ABCA1) and verified them using RT-qPCR. Functional enrichment analysis revealed that these genes were primarily associated with inflammatory response, chemokine production, and cellular energy metabolism. Further, we identified candidate drugs as potential treatments for LDH. Additionally, in immune infiltration analysis, the abundance of activated dendritic cells, neutrophils, and gamma delta T cells varied significantly between the LDH group and Control group. The scRNA-seq analysis showed that these hub genes were mainly expressed in chondrocyte-like cells. **Conclusions**: The identified EMERDEG hub genes and pathways offer novel insights into the molecular mechanisms underlying LDH and suggest potential therapeutic targets.

## 1. Introduction

Lumbar disc herniation (LDH) is a prevalent condition that represents a major cause of lower back pain and disability worldwide. The economic burden of this condition extends beyond the patients themselves, impacting healthcare systems globally [1]. Current treatment modalities for LDH include conservative management strategies, physical therapy, and surgical interventions. However, these approaches often exhibit limitations, including inconsistent efficacy and potential complications, necessitating the exploration of novel therapeutic targets to improve patient outcomes [2,3]. The causes and pathogenesis of this condition are multifaceted, involving both mechanical and biological factors [4].

Recent research has highlighted the critical roles of energy metabolism pathways and regulated cell death mechanisms [5], such as apoptosis and efferocytosis, in the degenerative processes of intervertebral discs. These biological processes not only support the survival and functionality of disc cells but also play pivotal roles in cellular responses to stress and injury. The nucleus pulposus (NP), the gel-like center of the intervertebral disc (IVD), is particularly sensitive to metabolic disturbances. IVD cells, particularly those in the NP and annulus fibrosus (AF), have unique energy demands that are critical for their function and maintenance. These cells rely heavily on aerobic metabolism to generate adenosine triphosphate (ATP), which is essential for various cellular processes, including matrix synthesis and cellular repair mechanisms [6]. The avascular nature of the IVD limits nutrient supply, making efficient energy metabolism crucial for cell survival and function [7,8,9,10]. Abnormalities in energy metabolism are closely linked to the process of apoptosis in IVD cells, particularly in the context of herniation and degeneration.

Studies have shown that IVD cells exhibit a high dependency on mitochondrial respiration [11,12], which is vital for maintaining homeostasis and responding to mechanical stressors. Disruption of energy metabolism can trigger cellular senescence and apoptosis, exacerbating disc degeneration. When IVD cells experience energy deficits, often due to mitochondrial dysfunction, they become more susceptible to apoptotic signaling pathways [13]. Meanwhile, factors such as oxidative stress, which is heightened in the degenerative disc environment, can compromise mitochondrial integrity and promote cell death [14]. Apoptotic cell debris can further promote inflammation by triggering immune responses and increasing oxidative stress [15].

In recent years, more and more studies have shown that inflammation at the injury site can hinder mitochondrial energy metabolism, leading to a lack of regeneration in the AF cells, which are crucial for disc integrity [16]. IVDs are typically considered immune-privileged sites. However, damage to the physical barriers between the NP and the systemic circulation can expose the NP to immune responses. This exposure can lead to chronic inflammation, characterized by the infiltration of various immune cells, including macrophages and T cells, which further contribute to the degeneration process [17]. Inflammatory cytokines released during this immune response can also perpetuate pain and promote further degeneration of the disc tissue.

The balance between cell survival and death is crucial for maintaining disc health, and disturbances in this balance can accelerate degeneration. Modulating the apoptotic pathways may offer a therapeutic approach to preserve disc function and prevent herniation. One such repair mechanism is efferocytosis—the process by which apoptotic cells are cleared by both professional and non-professional phagocytic cells [18]. Efficient efferocytosis is essential for resolving inflammation and restoring tissue homeostasis. The presence of apoptotic cells in the IVD can trigger further inflammation if not efficiently cleared through efferocytosis. Ineffective clearance of these cells can lead to secondary necrosis, which exacerbates the inflammatory response and contributes to the progression of disc degeneration [19,20].

Therefore, enhancing mitochondrial energy metabolism and efferocytosis function in IVD cells holds great promise as a potential therapeutic approach for LDH. Understanding how energy metabolism intersects with efferocytotic activity [21] could provide novel insights into the resolution of apoptosis and inflammation in LDH. However, the specific roles of energy metabolism and efferocytosis-related genes in the context of LDH have not been characterized. To fill these gaps, this study aims to comprehensively investigate the profiles of genes related to energy metabolism and efferocytosis within the context of LDH. The study may reveal novel therapeutic targets and intervention pathways by elucidating the interactive mechanisms of these genes in the regulation of apoptosis and inflammatory processes.

## 2. Materials and Methods

### 2.1. Data Download

Bulk-RNA sequencing data GSE124272 [22] and GSE150408 [23] and scRNA-seq sequencing data GSE230809 [24] were downloaded from the Gene Expression Omnibus (GEO) database (https://www.ncbi.nlm.nih.gov/geo/, (accessed on 1 December 2024)) using the keywords “Intervertebral Disc” (For detailed dataset Information, please refer to Supplementary Table S1). The GeneCards database (https://www.genecards.org/, (accessed on 1 December 2024)) provides comprehended information on human genes. Using “Energy Metabolism” or “Efferocytosis” as search keywords and retaining only “Protein Coding” genes, we obtained a total of 5941 energy metabolism-related genes (EMRGs) and 113 efferocytosis-related genes (ERGs). In addition, using “Energy Metabolism” or “Efferocytosis” as keywords to search for energy metabolism-related gene sets and efferocytosis-related gene sets in the published literature on the PubMed website (https://pubmed.ncbi.nlm.nih.gov/, (accessed on 1 December 2024)), we obtained 22 EMRGs [25] and 272 ERGs [26,27]. In this study, the EMRGs and ERGs obtained from the two sources were combined, resulting in 5941 EMRGs and 279 ERGs. Finally, by intersecting the 5941 EMRGs and 279 ERGs, we identified a total of 128 energy metabolism and efferocytosis-related genes (EMERGs, Supplementary Table S2).

### 2.2. Differential Expression Analysis

The sva R package (Version 3.50.0) [28] was used to remove batch effects from the GSE124272 and GSE150408 datasets, resulting in the combined GEO dataset. This combined dataset consists of 25 LDH samples and 25 Control samples. Subsequently, the limma R package (Version 3.58.1) [29] was applied for normalization, probe annotation, and standardization of the combined GEO dataset. To evaluate the effectiveness of batch effect removal, principal component analysis (PCA) was performed on the expression matrix before and after batch correction.

Differential expression analysis between the LDH and Control groups was conducted using the limma R package (Version 3.58.1). Genes with |logFC| > 0 and *p*-value < 0.05 were identified as differentially expressed genes (DEGs). Among these, genes with logFC > 0 and *p*-value < 0.05 were classified as upregulated genes, while those with logFC < 0 and *p*-value < 0.05 were classified as down-regulated genes. The results were visualized using a volcano plot generated by the ggplot2 R package (Version 3.4.4).

To identify energy metabolism and efferocytosis-related differentially expressed genes (EMERDEGs) associated with LDH, the intersection between all DEGs (|logFC| > 0, *p*-value < 0.05) from the combined GEO dataset and EMERGs was taken. A Venn diagram was plotted to visualize the overlap, and the EMERDEGs were displayed using a heatmap generated by the pheatmap R package (Version 1.0.12). Additionally, the RCircos R package (Version 1.2.2) [30] was used to generate a chromosome location map of the EMERDEGs.

To further investigate the expression differences of key genes in the LDH and Control groups, a group comparison plot was created based on the expression levels of EMERDEGs in the combined GEO dataset. EMERDEGs showing significant differential expression were selected for subsequent analyses.

### 2.3. Construction of Diagnostic Model for LDH

In order to find the key EMERDEGs that are closely related to LDH, we try to use machine learning methods to screen from gene expression data. This study employed three machine learning algorithms—univariate logistic regression, support vector machine (SVM) [31], and LASSO (least absolute shrinkage and selection operator) regression—to identify key genes associated with LDH. For univariate logistic regression analyses, *p*-value < 0.05 was considered the cutoff for the significant correlation. Then, the EMERDEGs included in the univariate logistic regression model were used to construct an SVM model through the SVM algorithm. Finally, LASSO regression analysis was performed using the R package glmnet (Version 4.1–8) [32] based on the EMERDEGs included in the SVM model. The LASSO risk score (RiskScore) was calculated based on the risk coefficient of the LASSO regression analysis. The risk score was calculated by the following formula:

$$RiskScore=\sum_{i}Coefficient\;\left({gene}_{i}\right)\;*\;mRNA\;Expression\;({gene}_{i})$$

### 2.4. Construction of Protein–Protein Interaction (PPI) Network and Regulatory Network

STRING (https://cn.string-db.org/, (accessed on 20 December 2024)) was used to predict interactions between proteins [33]. In this study, we utilized the STRING database to construct a PPI network based on key genes; we used the medium interaction score > 0.4 as a threshold. The PPI network was then visualized using Cytoscape v3.7.1 software. Additionally, it was employed to construct interaction networks and to make predictions regarding hub genes. The GeneMANIA website (https://genemania.org/, (accessed on 20 December 2024)) was utilized to predict genes with functions similar to those of the target genes [34].

The miRNAs associated with hub genes were obtained from the StarBase v3.0 database (https://starbase.sysu.edu.cn/, (accessed on 20 December 2024)), [35] and the mRNA-miRNA regulatory network was visualized using Cytoscape software.

### 2.5. Animal Model

The animal model of IVD disease was developed according to a previous method described [36,37]. A total of twenty male C57BL/6 mice, aged 3 months, were utilized in this research. They were randomly divided into two groups: a Control group (*n* = 10) and an LDH group (*n* = 10). All animal experiments were carried out with the approval of the Institutional Animal Care and Ethics Committee of the Fifth Affiliated Hospital of Sun Yat-sen University. Once the mice in the LDH group reached 3 months of age, we suspended their tails in specialized cages for a duration of 4 weeks, allowing the mice to eat freely in order to develop a model of intervertebral disc disease. The height of the suspension was adjusted to ensure that the hind legs of the mice were slightly elevated above the ground. After four weeks, we proceeded to collect the spines of the mice for further experiments.

### 2.6. Quantitative Reverse Transcription Polymerase Chain Reaction (RT-qPCR)

Total RNA was isolated from mouse intervertebral discs (L3–L6) using an RNA extraction kit (Solarbio, Beijing, China) following the manufacturer’s protocol. The total RNA was then reversely transcribed into cDNA by an iScript™ cDNA Synthesis kit (Bio-Rad, Hercules, CA, USA), and quantitative PCR reactions were performed with SsoAdvanced Universal SYBR Green Supermix (Bio-Rad, USA). RT-qPCR was performed using a CFX96 Real-Time PCR System (Bio-Rad, USA). The changes in gene expression were calculated using the 2^−ΔΔCt^ method, and the house-keeping gene β-actin was used as the endogenous control for normalization. The RT-PCR primers are shown in Supplementary Table S3.

### 2.7. Gene Ontology (GO) and Kyoto Encyclopedia of Genes and Genomes (KEGG)

We used the R package clusterProfiler (Version 4.10.0) [38] to perform GO and pathway (KEGG) enrichment analysis of hub genes. The item screening criteria were adj.*p* < 0.05 and FDR value (q value) < 0.235. The *p*-value correction method was Benjamini–Hochberg (BH).

### 2.8. Gene Set Enrichment Analysis (GSEA)

In this study, we first ranked the genes from the combined GEO datasets based on their log fold change (logFC) values between the LDH group and the Control group. We then performed Gene Set Enrichment Analysis (GSEA) [39] on all genes using the R package clusterProfiler. The parameters for GSEA were set as follows: seed = 2020, minimum gene set size = 10, and maximum gene set size = 500. The gene set ‘c2.all.v2023.2.Hs.symbols’ was acquired from the MSigDB database. Significant gene sets were identified based on adjusted *p*-values (adj.*p*) < 0.05 and false discovery rate (FDR or q-value) < 0.25, with *p*-value adjustment performed using the Benjamini–Hochberg (BH) method.

### 2.9. Immune Infiltration Analysis

CIBERSORT immune infiltrating analysis was performed to calculate the abundance of 22 immune infiltrating cells [40]. The gene expression matrix data were uploaded to CIBERSORT, samples with a *p*-value less than 0.05 were filtered out, and an immune cell infiltration matrix was generated. Subsequently, the correlation between immune cells was calculated based on the Spearman algorithm, and the R package pheatmap (Version 1.0.12) was used to draw the correlation heatmap to display the correlation analysis results of immune cells themselves. The correlation between hub genes and immune cells was calculated based on the Spearman algorithm. The R package ggplot2 (Version 3.4.4) was used to create a correlation bubble plot to visualize the analysis results of hub genes and immune cells.

### 2.10. Prediction of Hub Gene-Targeted Drugs

In this research, the Drug Signatures Database (DSigDB) was utilized to identify potential drugs that interact with the hub genes, aiming to offer guidance for targeted disease therapies [41]. Access to DSigDB is acquired through the Enrichr platform (https://maayanlab.cloud/Enrichr/, (accessed on 7 January 2025)). The candidate drugs were sorted by the adjusted *p*-value from small to large, and the adjusted *p*-value < 0.01 was considered statistically significant.

### 2.11. scRNA-seq Analysis

First, we used the “CreateSeuratObject” function from the R package Seurat v4.0 [42] to import the Counts matrix of the single-cell (scRNA-seq) dataset GSE230809 and create a Seurat object, setting the parameter to include cells with at least 200 expressed genes. The proportion of mitochondrial genes in the total genetic material may indicate whether a cell is in a steady state. Generally, when the proportion of mitochondrial genes in a cell is high relative to all genes, the cell may be under stress. Therefore, we filtered out cells with mitochondrial gene content >5%.

Next, we normalized the scRNA-seq dataset GSE230809 using the “NormalizeData” function. We then applied PCA to identify significant principal components (PCs) and visualized the *p*-value distribution using the “ElbowPlot” function. Finally, we selected 15 PCs for uniform manifold approximation and projection (UMAP) analysis for dimensionality reduction. We used the “FindNeighbors” function with default parameters and 15 PC dimensions to construct the k-nearest neighbor (KNN) graph based on the Euclidean distance in PCA space. By calling the “FindClusters” function and applying the “clustree” function, we determined a resolution of 1.5 to classify cells into different clusters. Finally, we used the “RunUMAP” function to perform dimensionality reduction, allowing visualization and exploration of the dataset.

Additionally, we annotated and identified cell types in the scRNA-seq dataset GSE230809 using the “SingleR” function from the R package SingleR (version 2.4.1) [43], based on the celldex package.

## 3. Results

### 3.1. Technology Roadmap

**Figure 1:**
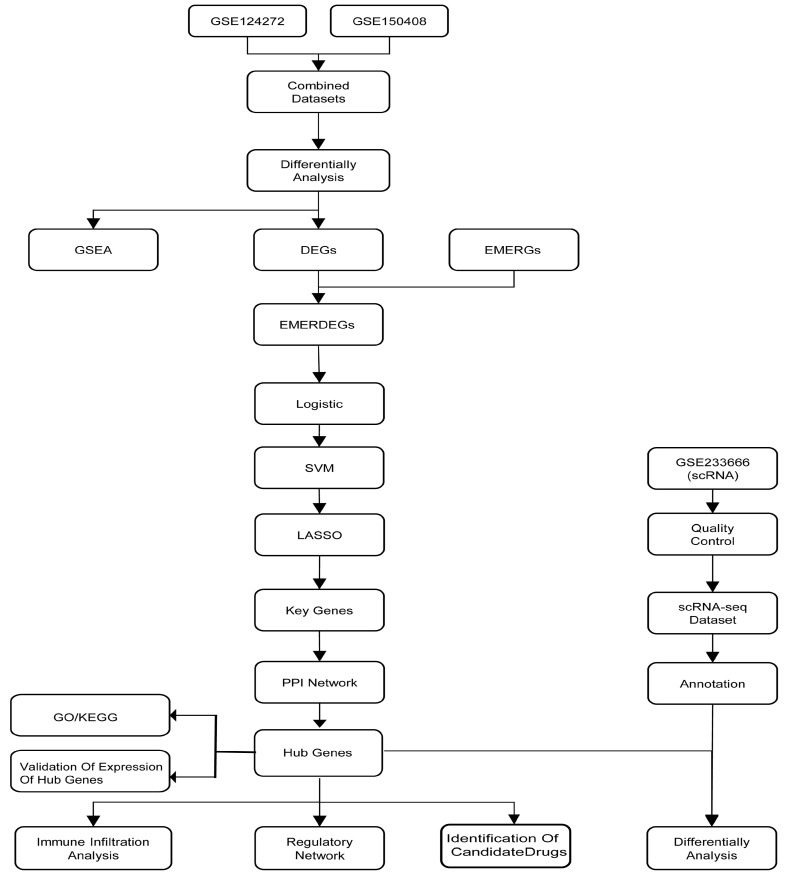
Technology roadmap.

The roadmap (Figure 1) below summarizes the main analytical steps used to identify hub genes and candidate drugs through integrated multi-omics analysis.

### 3.2. Merging of LDH Datasets

**Figure 2:**
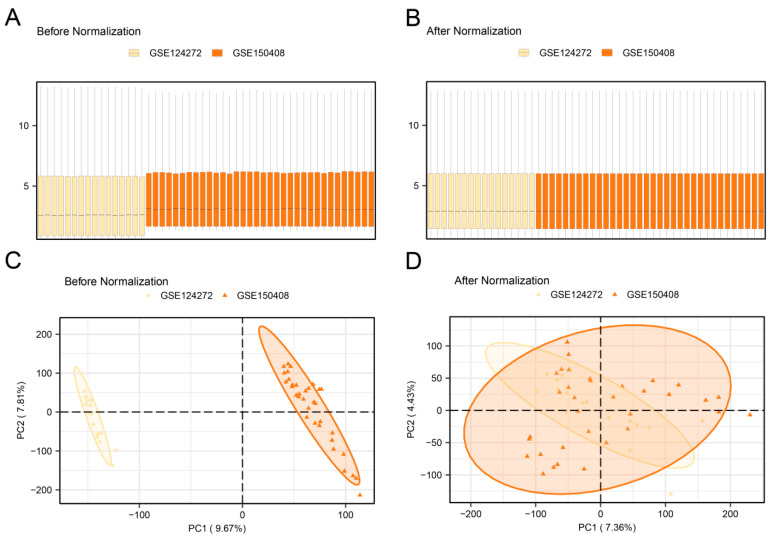
Batch effects removal of GSE124272 and GSE150408. (**A**) Boxplots of combined dataset distribution before batch removal. (**B**) Post-batch integrated combined dataset distribution boxplots. (**C**) PCA plot of the integrated combined dataset before debatching. (**D**) PCA plot of the integrated combined dataset after debatching.

First, the R package sva was used to remove batch effects in the LDH datasets GSE124272 and GSE150408 to obtain the combined dataset. Subsequently, boxplots (Figure 2A,B) were used to compare the expression values of the datasets before and after batch effect removal. Second, PCA (principal component analysis) plots (Figure 2C,D) were used to compare the distribution of low-dimensional features before and after batch effect correction. The results from the boxplots and PCA plots showed that batch effects in the LDH dataset were largely eliminated following correction.

### 3.3. Differentially Expressed Genes Related to Energy Metabolism and Efferocytosis in Patients with LDH

**Figure 3:**
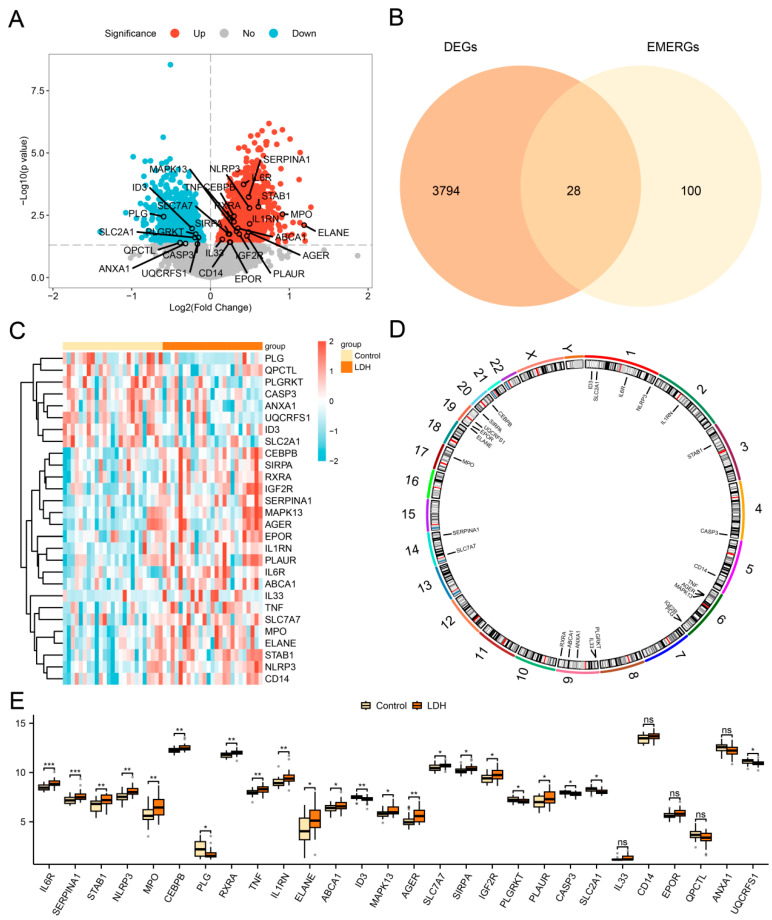
Differential gene expression analysis. (**A**) Volcano map of DEG analysis between LDH and Control group in the combined dataset. (**B**) Venn diagram illustrating the overlap between DEGs and EMERGs. (**C**) Heat map of EMERDEGs in the combined dataset. (**D**) Chromosomal mapping of EMERDEGs. (**E**) A comparison chart presents EMERDEGs in the combined dataset (*n* = 25/group, * *p* < 0.05, ** *p* < 0.01 and *** *p* < 0.001 vs. Control group; ns = not significant (*p* > 0.05)).

A total of 3822 differentially expressed genes (DEGs) meeting the threshold of |logFC| > 0 and *p*-value < 0.05 were identified in the combined dataset. Of these, 1882 genes were upregulated (logFC > 0, *p*-value < 0.05), and 1940 genes were downregulated (logFC < 0, *p*-value < 0.05). A volcano plot was generated based on the differential analysis results (Figure 3A).

In order to obtain the DEGs related to energy metabolism and efferocytosis (EMERDEGs), we intersected all DEGs (|logFC| > 0, *p*-value < 0.05) with genes associated with energy metabolism and efferocytosis (EMERGs) and generated a Venn diagram (Figure 3B). A total of 28 EMERDEGs were identified: *IL6R*, *SERPINA1*, *STAB1*, *NLRP3*, *MPO*, *CEBPB*, *PLG*, *RXRA*, *TNF, IL1RN*, *ELANE*, *ABCA1*, *ID3*, *MAPK13*, *AGER*, *SLC7A7*, *SIRPA*, *IGF2R*, *PLGRKT*, *PLAUR*, *CASP3*, *SLC2A1*, *IL33*, *CD14*, *EPOR*, *QPCTL*, *ANXA1*, and *UQCRFS1*. A heatmap (Figure 3C) was generated to visualize the expression patterns of these 28 EMERDEGs. We annotated their positions and created a chromosome location map to examine the locations of these 28 EMERDEGs on human chromosomes (Figure 3D). This map reveals that genes *ID3*, *SLC2A1*, *IL6R*, and *NLRP3* are located on chromosome 1; *PLGRKT*, *IL33*, *ANXA1*, *ABCA1*, and *RXRA* are located on chromosome 9; while *TNF*, *AGER*, *MAPK13*, *IGF2R*, and *PLG* are located on chromosome 6. The remaining genes are dispersed across various chromosomes.

Finally, we examined the variations in the expression levels for 28 EMERDEGs between the LDH and Control groups within the combined dataset. Differential analysis identified 23 EMERDEGs with significantly different expression levels between the LDH and Control groups (*p* < 0.05). These 23 EMERDEGs were included in subsequent analyses.

### 3.4. Construction of Diagnostic Model for Lumbar Disc Herniation

**Figure 4:**
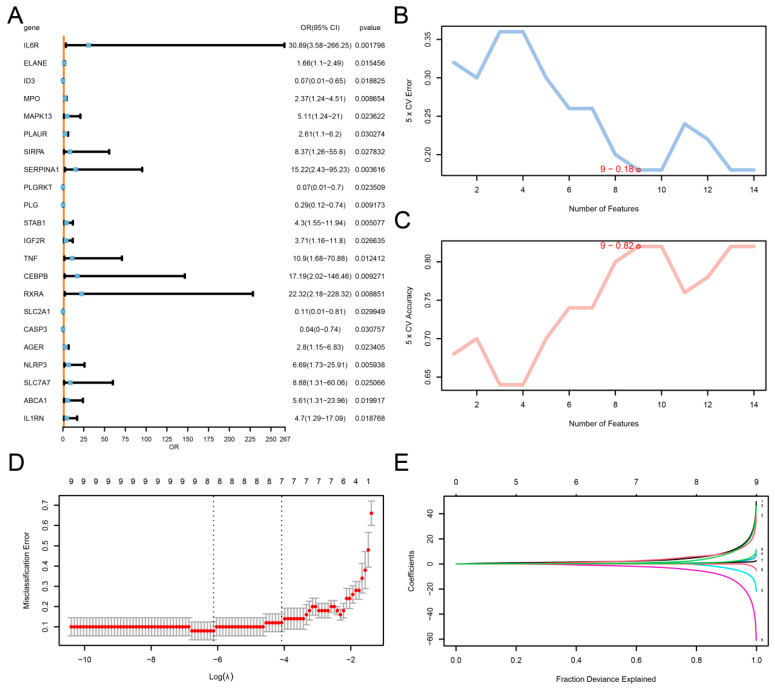
Diagnostic model of LDH. (**A**) Forest plot lines of significant key genes related to LDH using univariate logistic regression. SVM algorithm to obtain the error rate of the minimum number of genes (**B**) and the highest accuracy of the number of genes (**C**). Diagnostic model plot (**D**) and variable trajectory plot (**E**) of LASSO regression model.

To evaluate the diagnostic potential of the 23 EMERDEGs in LDH, a univariate logistic regression model was constructed. All 23 EMERDEGs showed statistically significant associations with LDH (*p*-value < 0.05), as visualized in the forest plot (Figure 4A). Subsequently, an SVM (support vector machine) model was constructed based on these 23 EMERDEGs, identifying the number of genes with the lowest error rate (Figure 4B) and the highest accuracy (Figure 4C). The results indicated that when the number of genes was nine, the SVM model achieved the highest accuracy. These nine genes were *IL6R*, *TNF*, *MAPK13*, *ELANE*, *PLAUR*, *ID3*, *ABCA1*, *RXRA*, and *STAB1*. Finally, a LASSO regression model was applied to the nine SVM-selected genes to refine the diagnostic signature. The LASSO coefficient profiles (Figure 4D) and variable trajectory plot (Figure 4E) revealed eight key genes *(IL6R*, *TNF*, *MAPK13*, *ELANE*, *PLAUR*, *ID3*, *ABCA1*, and *STAB1*) as the most predictive features for LDH diagnosis.

### 3.5. Protein–Protein Interaction (PPI) Network and Regulatory Network

**Figure 5:**
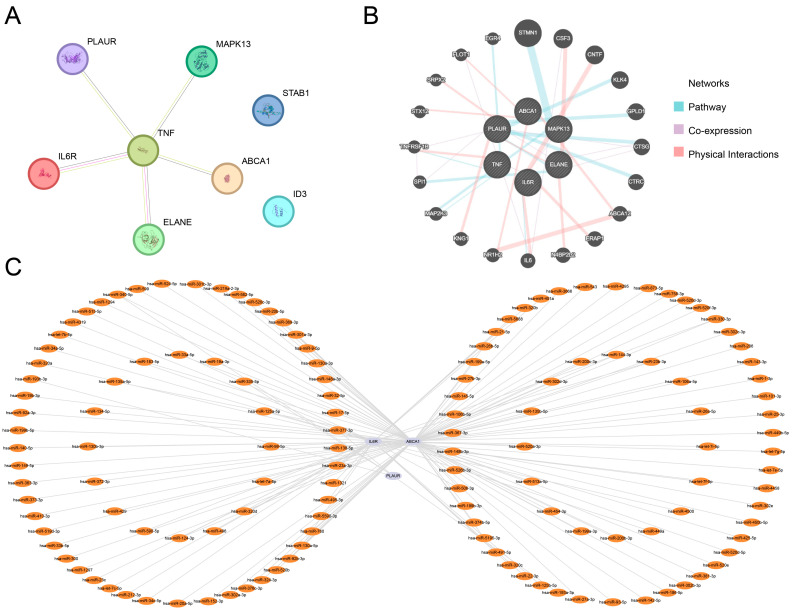
PPI network and regulatory network. (**A**) PPI network of key genes constructed using the STRING database. (**B**) Interaction network of hub genes and functionally similar genes predicted by the GeneMANIA database. In the figure, circles represent hub genes and functionally similar genes, while different line colors indicate various functional relationships. (**C**) mRNA-miRNA regulatory network of hub genes. Light purple represents mRNAs, and orange denotes miRNAs.

First, the PPI network of the eight key genes was constructed using the STRING database (Figure 5A). The results indicated that six genes—*IL6R*, *TNF*, *MAPK13*, *ELANE*, *PLAUR*, and *ABCA1*—were interconnected. These six genes were defined as hub genes.

Next, the GeneMANIA database was used to predict and construct an interaction network of the six hub genes along with their functionally similar genes (Figure 5B). Different colored lines in the network represented co-expression, shared protein domains, and other interactions. The network included the six hub genes as well as 20 functionally similar proteins.

Finally, miRNAs associated with the hub genes were obtained from the StarBase database, and an mRNA-miRNA regulatory network was constructed and visualized using Cytoscape software (Figure 5C). This network included three hub genes and 135 miRNAs, with detailed information provided in Table S4.

### 3.6. Animal Models, IVD RNA Extraction, and RT-qPCR

**Figure 6:**
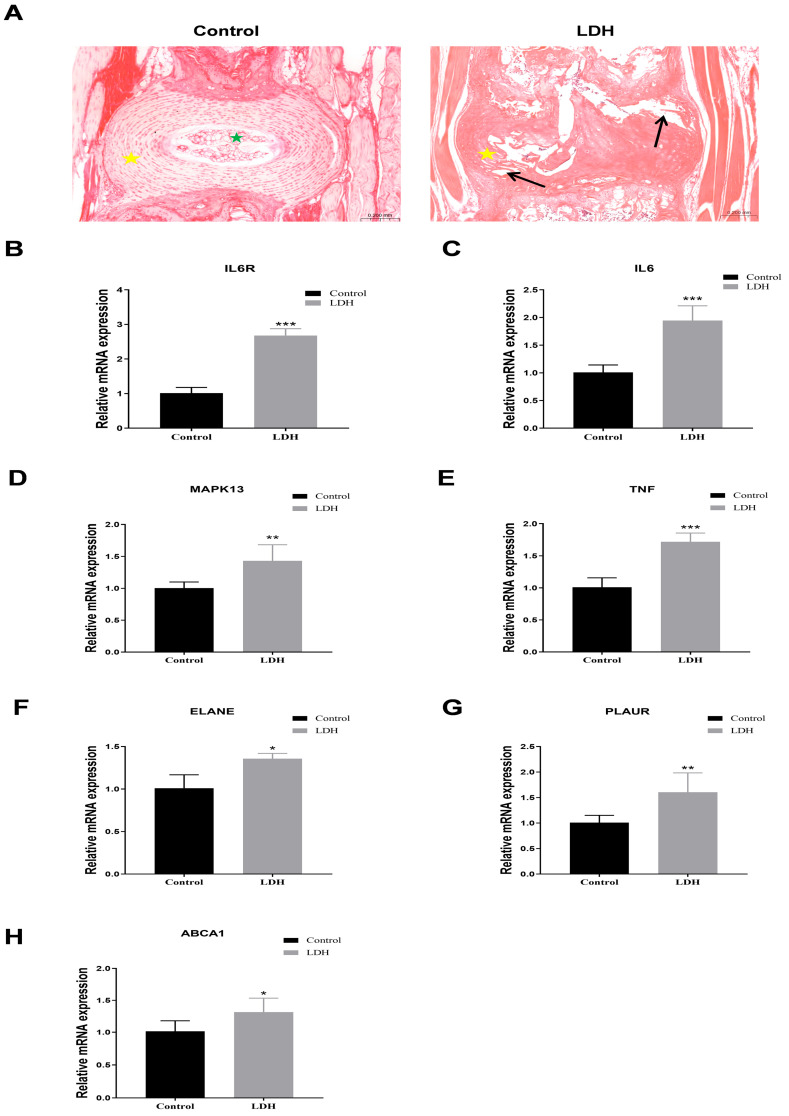
Validation of the expression of hub genes. (**A**) Representative images of H&E staining of IVD in mice from different groups. The Control group was characterized by an intact structure and a clear distinction between the outer fibrous annulus (yellow pentagram) and inner nucleus pulposus (green pentagram). The LDH group was characterized by structural disorganization, loss of boundary between fibrous annulus and nucleus pulposus, and fibrous annulus rupture (black arrow). (**B**–**H**) RT-qPCR showed the mRNA expression level of hub genes in IVD tissues of mice in each group (*n* = 10/group). * *p* < 0.05, ** *p* < 0.01, *** *p* < 0.001.

To further validate the important role of hub genes in LDH, we employed the IVD degeneration mouse model. The detection of hub genes showed that, compared to the Control group, the expression of *IL6R* and its ligand *IL6*, as well as *TNF*, *MAPK13*, *ELANE*, *PLAUR*, and *ABCA1*, was significantly increased in the IVD tissue of the LDH group (Figure 6B–G).

### 3.7. Gene Ontology (GO) and Pathway (KEGG) Enrichment Analysis

**Figure 7:**
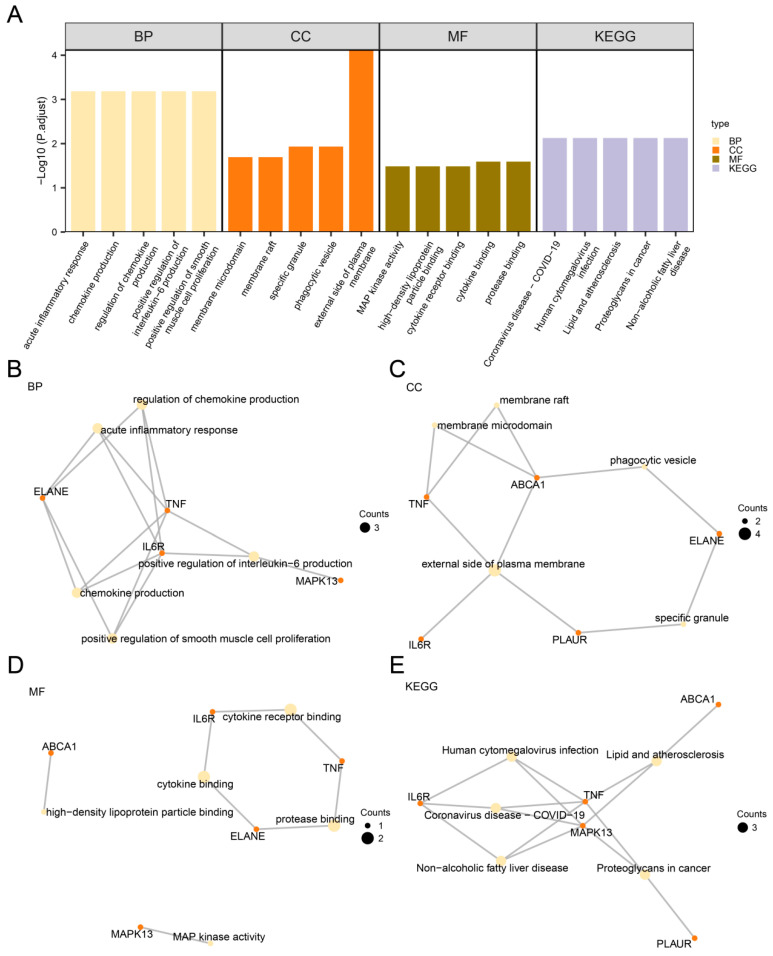
GO and KEGG enrichment analysis for hub genes. (**A**) Bar chart depicting the results of GO and KEGG pathway enrichment analyses of hub genes, categorized into biological processes (BP), cellular components (CC), molecular functions (MF), and KEGG pathways. The x-axis represents GO and KEGG terms. (**B**–**E**) Network diagrams illustrating the GO and KEGG enrichment analysis results for hub genes, with panels corresponding to BP (**B**), CC (**C**), MF (**D**), and KEGG pathways (**E**). In these networks, light yellow nodes represent enriched terms, orange nodes denote hub genes, the size of the black nodes corresponds to the count value, with larger dots representing higher counts, and connecting lines indicate their relationships. The screening criteria for GO and KEGG enrichment analysis were adj.*p* < 0.05, FDR value (q value) < 0.235, and the *p*-value correction method was Benjamini–Hochberg (BH).

The GO and KEGG enrichment analyses were performed based on the six hub genes, with detailed results provided in Table S5. The GO and KEGG enrichment results were visualized in a bar plot (Figure 7). The findings indicate that these genes in LDH are predominantly enriched in the following biological processes (BPs): positive regulation of smooth muscle cell proliferation, positive regulation of interleukin-6 production, chemokine production and regulation, and acute inflammatory response.

For cellular components (CCs), the hub genes are primarily associated with the external side of the plasma membrane, phagocytic vesicle, specific granule, membrane raft, and membrane microdomain. Regarding molecular functions (MFs), they are involved in protease binding, cytokine binding, cytokine receptor binding, high-density lipoprotein particle binding, and MAP kinase activity.

Additionally, KEGG pathway enrichment analysis revealed significant associations with non-alcoholic fatty liver disease, proteoglycans in cancer, lipid, and atherosclerosis, human cytomegalovirus infection, and COVID-19.

### 3.8. GSEA Reveals Key Pathways

**Figure 8:**
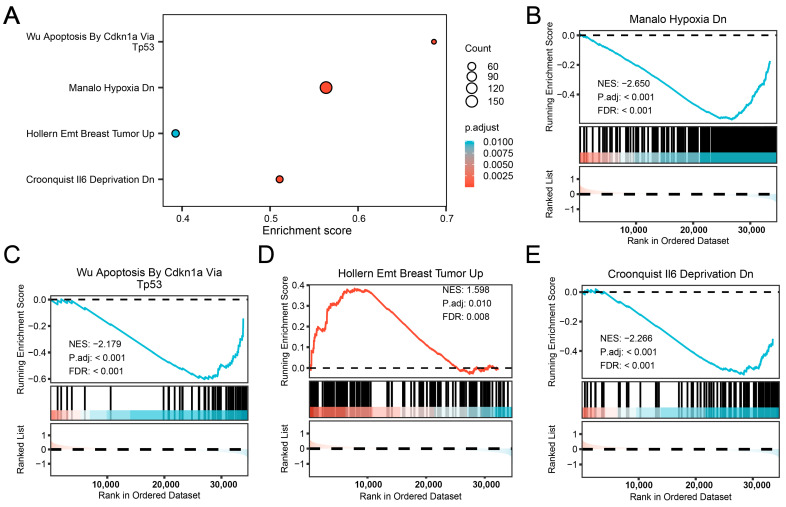
Gene set enrichment analysis. (**A**) A bubble plot of the GSEA. (**B**–**E**) GSEA showed that the combined dataset was significantly enriched in MANALO HYPOXIA DN (**B**), WU APOPTOSIS BY CDKN1A VIA TP53 (**C**), HOLLERN EMT BREAST TUMOR UP (**D**), and CROONQUIST IL6 DEPRIVATION DN (**E**). The screening criteria of GSEA enrichment screening were adjusted with *p*-value < 0.05 and FDR value (q-value) < 0.25 as selection thresholds. The Benjamini–Hochberg method was applied for *p*-value correction.

We conducted GSEA to examine the influence of gene expression levels on the disparities between the LDH and Control groups in the combined dataset. The results were visualized using a bubble plot (Figure 8A), with detailed findings presented in Table S6. In the pathway map (Figure 8B–E), we present the significantly enriched pathways, including MANALO HYPOXIA DN (Figure 8B), WU APOPTOSIS BY CDKN1A VIA TP53 (Figure 8C), HOLLERN EMT BREAST TUMOR UP (Figure 8D), and CROONQUIST IL6 DEPRIVATION DN (Figure 8E).

### 3.9. Immune Infiltration Analysis (CIBERSORT)

**Figure 9:**
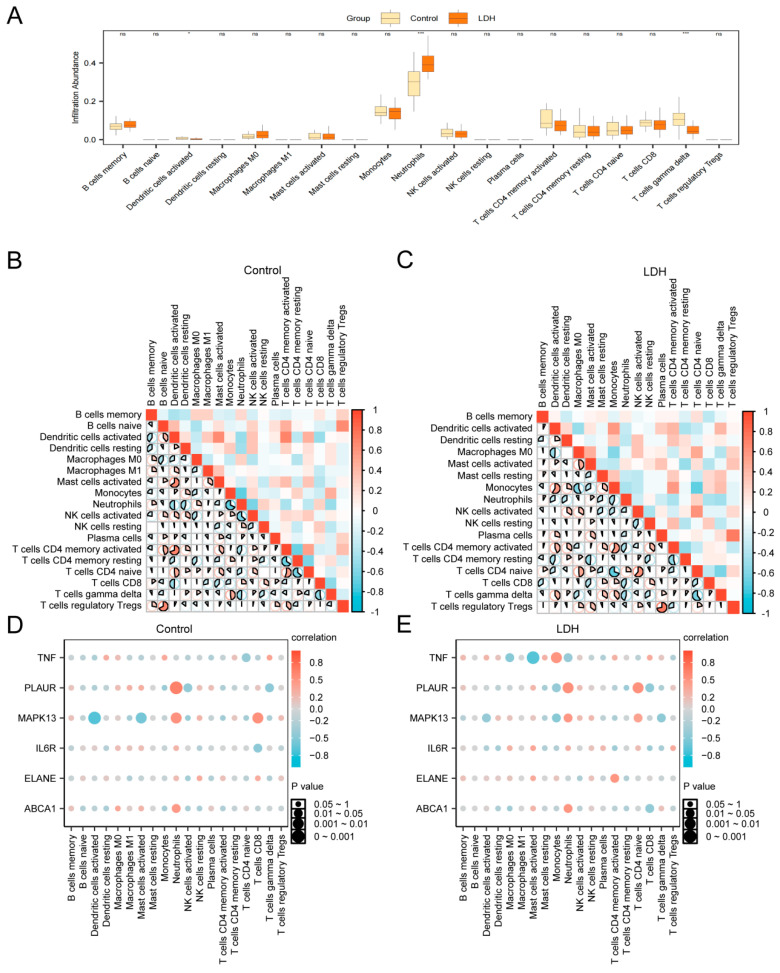
Immune infiltration analysis by CIBERSORT algorithm. (**A**) Box plots represent comparisons between the LDH group and the Control group in terms of the abundance of different immune cell infiltrates. (**B**,**C**) Correlation heatmaps of immune cells in the Control group (**B**) and LDH group (**C**) from the combined dataset. (**D**,**E**) Bubble plots showing the correlation between immune cell infiltration abundance and hub genes in the LDH group and the Control group from the combined dataset; * represents *p*-value < 0.05, statistically significant; *** represents *p*-value < 0.001 and highly statistically significant; ns represents not significant (*p* > 0.05). The absolute value of the correlation coefficient (r-value) below 0.3 represents weak or no correlation; between 0.3 and 0.5, a weak correlation; between 0.5 and 0.8, a moderate correlation; and above 0.8, a strong correlation. Red shows a positive correlation; blue shows a negative correlation. The depth of the color represents the strength of the correlation.

We employed the CIBERSORT algorithm to assess the abundance of 22 different immune cell types in the combined dataset and investigate the variation in immune infiltration between the LDH and Control groups. The results showed that the 19 immune cells were enriched in the combined dataset. Among them, activated dendritic cells, neutrophils, and gamma delta T cells showed significant differences between the LDH and Control groups, with neutrophils significantly elevated in the LDH group (Figure 9A).

To further investigate immune cell interactions, a correlation heatmap was generated to illustrate the relationships among the 19 immune cell types in both groups (Figure 9B,C). In the Control group, most immune cells exhibited strong correlations, with the strongest positive correlation observed between CD4 memory-activated T cells and activated dendritic cells (r = 0.692, *p* < 0.05) (Figure 9B). Similarly, in the LDH group, strong correlations were also observed, with plasma cells and regulatory T cells (Tregs) showing the highest positive correlation (r = 0.722, *p* < 0.05) (Figure 9C).

Additionally, correlation bubble plots were used to visualize the relationships between hub genes and immune cell infiltration (Figure 9D,E). In the Control group, MAPK13 exhibited the strongest negative correlation with activated dendritic cells (r = −0.704, *p* < 0.05) (Figure 9D), while in the LDH group, TNF showed the strongest negative correlation with activated mast cells (r = −0.693, *p* < 0.05) (Figure 9E).

### 3.10. Identification of Candidate Drugs

In the aspects of hub genes (*IL6R*, *TNF*, *MAPK13*, *ELANE*, *PLAUR*, and *ABCA1*) as potential drug targets in LDH, small-molecule compounds that may bind to these hub genes were identified using the DSigDB database. Table S7 shows the top 10 chemical compounds extracted based on their Adjusted *p*-value. Among these, the three leading candidate drugs are thalidomide, malondialdehyde, and 1,3-dimethylthiourea.

### 3.11. Analysis of Cell-Specific Expression of Hub Genes

**Figure 10:**
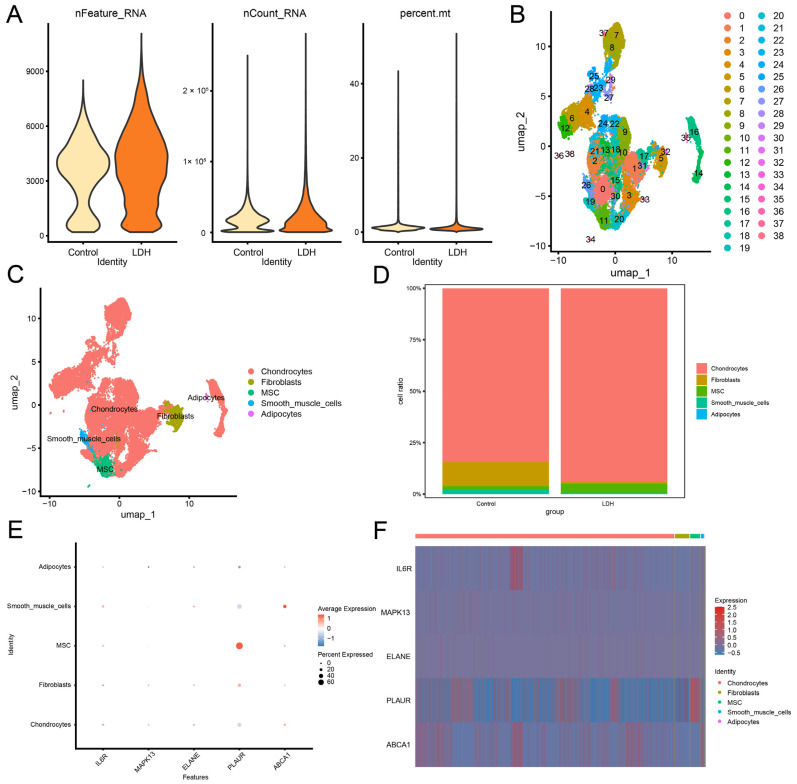
Quality control and cell annotation of single-cell datasets. (**A**) GSE230809 single-cell dataset quality control nCount_RNA, nFeature_RNA, mitoRatio violin plot. (**B**) UMAP clustering plot of 39 cell clusters in the GSE230809 dataset. (**C**) UMAP clustering map of 5 different cell groups in the GSE230809 dataset. (**D**) Stacking bar plot of five different cell groups in the GSE230809 dataset. Dot plot (**E**) and heat map (**F**) of expression values of hub genes in different cell types. UMAP, uniform manifold approximation and projection.

Following the Seurat analysis workflow, we first conducted quality control on the single-cell dataset GSE230809 (Figure 10A) and determined the optimal number of principal components to be 15 using the Elbow method. Through unsupervised clustering, a total of 39 distinct cell subsets were identified and visualized using the UMAP method (Figure 10B). Subsequently, manual annotation classified these cell clusters into five major cell groups—adipocytes, smooth muscle cells, mesenchymal stem cells (MSCs), fibroblasts, and chondrocytes—also visualized via UMAP (Figure 10C). Chondrocyte-like cells were the main cells in the IVD.

The proportional composition of these cell types in the LDH and Control groups was analyzed and is depicted in Figure 10D. Additionally, a dot plot (Figure 10E) and a heatmap (Figure 10F) were generated to illustrate the expression patterns of hub genes across the five cell types. The GSE230809 dataset contains five hub genes, including *IL6R*, *MAPK13*, *ELANE*, *PLAUR*, and *ABCA1*. Analysis of hub gene expression revealed that *IL6R*, *MAPK13*, and *ELANE* were predominantly expressed in chondrocytes, *PLAUR* exhibited high expression in MSCs, and *ABCA1* was relatively highly expressed in smooth muscle cells.

## 4. Discussion

LDH is a prevalent condition that significantly impacts patients’ quality of life and imposes substantial economic burdens on healthcare systems. Current treatment modalities, including conservative management, physical therapy, and surgical interventions, often exhibit limitations such as inconsistent efficacy and potential complications. Therefore, exploring novel therapeutic targets is imperative to enhance patient outcomes. In light of these challenges, our research focuses on elucidating the roles of energy metabolism and efferocytosis in the context of LDH. Efferocytosis not only prevents tissue necrosis and inflammation caused by the secondary necrosis of dying cells but also facilitates pro-resolving signaling within macrophages, a process vital for tissue resolution and repair after injury or inflammation [44]. The identification of EMERDEGs and their associated pathways provides a foundation for developing targeted therapeutic strategies.

The investigation of energy metabolism pathways and regulated cell death mechanisms, particularly apoptosis and efferocytosis, is crucial in understanding the pathophysiology of LDH. Recent research highlights a significant correlation between metabolic dysregulation and the apoptotic processes occurring within IVD cells. The reliance of IVD cells on energy or ATP production underlines the importance of energy homeostasis for their survival and functionality [45]. When energy deficits arise, often due to mitochondrial impairments, cells become increasingly susceptible to apoptosis, indicating a critical link between bioenergetics and cell death pathways. This interplay suggests that therapeutic strategies targeting energy metabolism could potentially alter the course of disc degeneration and improve patient outcomes [46]. Furthermore, the failure of effective efferocytosis following cellular apoptosis may exacerbate inflammation, leading to further degeneration of the disc tissue. Understanding how these mechanisms interact provides valuable insights into the development of novel therapeutic interventions aimed at preserving disc integrity and function.

We identified 3822 DEGs in LDH patients, with 1882 upregulated and 1940 downregulated. Notably, six EMERDEGs, including *IL6R*, *TNF*, *MAPK13*, *ELANE*, *PLAUR*, and *ABCA1*, were highlighted. Elevated *IL6R* expression may reflect enhanced cellular responsiveness to IL-6 signaling. For instance, IL6 and TNF are well-documented mediators of inflammation and pain in disc degeneration. IL6 can activate downstream signaling pathways that contribute to inflammation and catabolic processes within the IVD, potentially leading to degeneration [47]. TNF can exacerbate disc degeneration by promoting the expression of matrix metalloproteinases (MMPs) and other catabolic enzymes that degrade the extracellular matrix of the disc [48]. MAPK13 is part of the mitogen-activated protein kinase (MAPK) family, which is involved in cellular responses to stress and inflammation [49]. ABCA1, an ATP-binding cassette transporter, is known for its role in cholesterol efflux and lipid homeostasis. Its expression can be influenced by inflammatory cytokines such as TNF [50]. Overall, these molecules are integral to the complex network of inflammation and may contribute to IVD degeneration.

Enrichment analysis revealed that hub genes were primarily involved in biological processes such as the responses to IL-6 signaling and acute inflammatory response. Pathways like non-alcoholic fatty liver disease and lipid and atherosclerosis were also implicated. The GSEA results revealed a high enrichment of genes in MANALO HYPOXIA DN, CROONQUIST IL6 DEPRIVATION DN, and WU APOPTOSIS BY CDKN1A VIA TP53. The NP of the IVD exists in a naturally hypoxic environment due to the lack of direct blood supply. The IVD is naturally avascular, resulting in a hypoxic microenvironment. Hypoxia-inducible factor-1α (HIF-1α) is a key regulator in this context, promoting extracellular matrix (ECM) synthesis and energy metabolism. However, during IVD degeneration, the expression of HIF-1α decreases, leading to impaired cellular function and ECM degradation. Hypoxia-induced downregulation of HIF-1α impairs cell survival and ECM production, leading to increased apoptosis. Apoptotic cell loss releases intracellular contents that can act as damage-associated molecular patterns (DAMPs), further stimulating inflammation. The resulting inflammatory environment exacerbates ECM degradation and promotes additional cell death, perpetuating disc degeneration [6,51]. This suggests a complex interplay between energy metabolic, apoptotic, and inflammatory pathways in LDH pathophysiology.

Normally, the IVD is an immune-privileged site due to its avascular nature and extracellular matrix composition. However, various pathological conditions can disrupt this immune privilege, leading to immune cell infiltration and inflammation. The infiltration of various immune cell types, such as activated dendritic cells and neutrophils, into the IVD environment can significantly influence inflammation and tissue repair [52]. The CIBERSORT analysis from our study revealed a distinct immune cell profile associated with LDH, indicating that neutrophils may contribute to the inflammatory milieu characteristic of this condition. Another study reported variations in immune infiltration profiles between LDH and Control groups, noting a higher proportion of regulatory T cells and macrophages in LDH tissues [53]. Moreover, the correlation between hub genes and immune cells was analyzed in our research. In the LDH group, the gene *PLAUR* exhibited a significant positive correlation with the neutrophil cells and naive CD4 T cells, while the *TNF* gene exhibited the strongest negative correlation with the immune Mast cells activated. Thus, identifying immune profiles in LDH patients may help tailor treatments.

Additionally, candidate drugs such as thalidomide and malondialdehyde were identified as potential treatments targeting hub genes associated with LDH. This drug discovery process introduces new avenues for therapeutic interventions, potentially leading to the improved management of LDH and its associated symptoms. The repurposing of existing drugs for LDH treatment has also been explored in other studies. For instance, research into the traditional Chinese medicine Yaobishu (YBS) identified compounds like palmitic acid that may act on LDH by regulating inflammatory and metabolic pathways [54]. Our study contributes to this field by identifying different candidate compounds, thereby expanding the potential therapeutic arsenal against LDH.

This study is subject to several limitations that warrant consideration. Firstly, the relatively small sample size could hinder the generalizability of our results and increase the risk of statistical bias. Second, potential biases may arise from integrating multiple datasets, which could impact the accuracy of the analyses. These limitations underscore the necessity for future studies to incorporate larger cohorts and experimental validation, thereby enhancing the credibility of the identified biomarkers and therapeutic targets. In addition, the hub genes identified through bulk and single-cell RNA sequencing dataset analyses were not further validated at the protein level due to the absence of Western blot or immunohistochemical staining experiments. This limits the confirmation of gene expression patterns and functional relevance at the protein level within lumbar disc tissues. Furthermore, although our analysis revealed potential immune cell subsets involved in efferocytosis and energy metabolism, we did not perform phenotypic validation of infiltrating immune cells using flow cytometry, immunostaining, or cell-type-specific markers. As such, the precise cellular sources and spatial localization of the identified genes remain speculative. Future studies will incorporate protein-level validation, spatial transcriptomics, and immune cell profiling to further elucidate the roles of these hub genes and immune populations in the pathophysiology of LDH.

In conclusion, our research elucidates the intricate interplay among energy metabolism, efferocytosis, and immune response in LDH. By identifying hub genes and establishing a diagnostic model, we highlight potential therapeutic targets that could significantly enhance patient care. The insights gained from this study may pave the way for novel interventions aimed at improving the management of LDH, ultimately contributing to better patient outcomes and reducing the economic burden associated with this condition. Future studies should focus on validating these findings through experimental approaches and exploring the clinical applicability of the proposed therapeutic strategies.

## Data Availability

Data and materials used to support the findings of this study are available from the corresponding author upon request.

## Supplementary Materials

The following supporting information can be downloaded at https://www.mdpi.com/article/10.3390/biomedicines13071536/s1: Supplementary Tables: Table S1. GEO dataset Information; Table S2. List of EMERGs; Table S3. Primer sequences for RT-qPCR; Table S4. List of mRNA-miRNA interaction network nodes; Table S5. Results of GO and KEGG Enrichment Analysis for hub genes; Table S6. Results of GSEA between LDH and Control group; Table S7. List of the suggested drugs for LDH.

## Abbreviations

The following abbreviations are used in this manuscript:

LDH	Lumbar disc herniation
IVD	Intervertebral disc
EMERDEGs	Energy metabolism and efferocytosis-related differentially expressed genes
EMERGs	Energy metabolism and efferocytosis-related genes
GSEA	Gene set enrichment analysis
PPI	Protein–protein interaction
NP	Nucleus pulposus
AF	Annulus fibrosus
