# Supplementary material for: Integrating Bulk RNA and Single-Cell Sequencing Data Reveals Genes Related to Energy Metabolism and Efferocytosis in Lumbar Disc Herniation
Source: Biomedicines. 2025 Jun 24;13(7):1536. doi: 10.3390/biomedicines13071536 (PMC12292327; doi:10.3390/biomedicines13071536)
Supplement: Supplementary file 1 [file biomedicines-13-01536-s001.zip › biomedicines-3633393-supplementary.pdf]

Table S1. GEO dataset Information

	GSE124272	GSE150408	GSE230809
Platform	GPL21185	GPL21185	GPL30173
Species	Homo sapiens	Homo sapiens	Homo sapiens
Tissue	Whole Blood	Whole Blood	Intervertebral disc
Samples in LDH group	8	17	6
Samples in Control group	8	17	18

GEO，Gene Expression Omnibus；LDH，Lumbar Disc Herniation。

Table S2. List of EMERGs

EMERGs				
MAP2K1	TLR3	TGM2	EGLN3	MPO
DNMT3A	CAMK2B	AGER	C3	ARG2
ITGB3	PTGS2	SERPINA1	CPT1B	DNM1L
FN1	C1QA	ABCA1	IL10	RXRA
CAMK2D	MAPK13	CRKL	IL1B	IGF2R
LIPA	THBS1	LGALS3	NR1H3	SLC16A1
CASP1	ALOX12	UBE2D3	SLC25A1	BCL2
ANXA1	SLC14A1	IL1RN	BSG	CEBPB
MAPKAPK2	IL33	IFNB1	SIRT6	ATP2A2
TYRO3	ACKR2	PLG	MAPK1	CREB1
IRF3	RAB17	UQCRFS1	CASP3	IL6
SIRPA	TGFBRAP1	CAMK2A	CPT1C	MTOR
SFTPD	PLGRKT	SIRPB1	NFE2L2	MAPK14
VTN	EPOR	NLRP3	QPCTL	CD36
ABCC11	CD274	ARNT	EPO	CPT1A
DYNLT1	CD47	PTPN11	NFATC4	NRF1
ID3	MAP2K2	CALR	ARG1	PPARD
STAB1	PLCG1	IL6R	CAMK2G	NR1H2
VPS16	HMGB1	MAPK3	GATA2	APOE
TGFB1	RAC1	RHOA	KNG1	GAPDH
CD14	MFGE8	RAB7A	ODC1	SLC2A1
CHI3L1	ANO6	TREM2	MEGF10	SIRT1

EMERGs				
PLAUR	GPR101	CRK	ANO5	HIF1A
CXCR4	LRP1	ALDH2	TNF	PPARG
ADAM17	CLU	ANXA2	SGK1	UCP2
ELANE	SLC7A7	JAK2		

EMERGs，Energy Metabolism-and Efferocytosis-Related Genes。

Table S3 .Primer sequences for RT-qPCR

Genes	Forward primer	Reverse primer
β-actin	CATTGCTGACAGGATGCAGAAGG	TGCTGGAAGGTGGACAGTGAGG
TNF	CTGAACTTCGGGGTGATCGG	GGCTTGTCACTCGAATTTTGAGA
IL6	TCTATACCACTTCACAAGTCGGA	GAATTGCCATTGCACAACTCTTT
IL6R	GGTGGCCCAGTACCAATGC	GGACCTGGACCACGTGCT
MAPK13	TCACTCGGAAAAGGGGCTTC	TTGTCGATGGCCGAGCAC
ELANE	CAGAGGCGTGGAGGTCATTT	GAAGATCCGCTGCACAGAGA
PLAUR	CGCCACAAACCTCTGCAAC	CTCTGTAGGATAGCGGCATTG
ACBA1	GCTTGTTGGCCTCAGTTAAGG	GTAGCTCAGGCGTACAGAGAT

Table S4. List of mRNA-miRNA interaction network nodes

mRNA	miRNA
ABCA1	hsa-miR-17-5p
ABCA1	hsa-miR-19a-3p
ABCA1	hsa-miR-19b-3p
ABCA1	hsa-miR-20a-5p
ABCA1	hsa-miR-25-3p
ABCA1	hsa-miR-26a-5p
ABCA1	hsa-miR-26b-5p
ABCA1	hsa-miR-27a-3p
ABCA1	hsa-miR-32-5p
ABCA1	hsa-miR-33a-5p
ABCA1	hsa-miR-92a-3p
ABCA1	hsa-miR-93-5p
ABCA1	hsa-miR-101-3p
ABCA1	hsa-miR-106a-5p
ABCA1	hsa-miR-199a-5p
ABCA1	hsa-miR-199a-3p
ABCA1	hsa-miR-148a-3p

mRNA	miRNA
ABCA1	hsa-miR-183-5p
ABCA1	hsa-miR-199b-5p
ABCA1	hsa-miR-200b-3p
ABCA1	hsa-miR-1-3p
ABCA1	hsa-miR-23b-3p
ABCA1	hsa-miR-27b-3p
ABCA1	hsa-miR-124-3p
ABCA1	hsa-miR-130a-3p
ABCA1	hsa-miR-135a-5p
ABCA1	hsa-miR-140-5p
ABCA1	hsa-miR-142-5p
ABCA1	hsa-miR-143-3p
ABCA1	hsa-miR-144-3p
ABCA1	hsa-miR-145-5p
ABCA1	hsa-miR-152-3p
ABCA1	hsa-miR-9-5p
ABCA1	hsa-miR-134-5p
ABCA1	hsa-miR-149-5p
ABCA1	hsa-miR-186-5p
ABCA1	hsa-miR-206
ABCA1	hsa-miR-200c-3p
ABCA1	hsa-miR-106b-5p
ABCA1	hsa-miR-302a-3p
ABCA1	hsa-miR-301a-3p
ABCA1	hsa-miR-130b-3p
ABCA1	hsa-miR-363-3p
ABCA1	hsa-miR-302b-3p
ABCA1	hsa-miR-302c-3p
ABCA1	hsa-miR-302d-3p
ABCA1	hsa-miR-367-3p
ABCA1	hsa-miR-376c-3p
ABCA1	hsa-miR-369-3p
ABCA1	hsa-miR-372-3p
ABCA1	hsa-miR-373-3p
ABCA1	hsa-miR-381-3p
ABCA1	hsa-miR-330-3p
ABCA1	hsa-miR-135b-5p
ABCA1	hsa-miR-148b-3p
ABCA1	hsa-miR-324-3p
ABCA1	hsa-miR-20b-5p
ABCA1	hsa-miR-429
ABCA1	hsa-miR-410-3p
ABCA1	hsa-miR-520e

mRNA	miRNA
ABCA1	hsa-miR-520f-3p
ABCA1	hsa-miR-520a-3p
ABCA1	hsa-miR-526b-3p
ABCA1	hsa-miR-520b
ABCA1	hsa-miR-520c-3p
ABCA1	hsa-miR-524-5p
ABCA1	hsa-miR-519d-3p
ABCA1	hsa-miR-520d-5p
ABCA1	hsa-miR-520d-3p
ABCA1	hsa-miR-513a-5p
ABCA1	hsa-miR-506-3p
ABCA1	hsa-miR-92b-3p
ABCA1	hsa-miR-582-5p
ABCA1	hsa-miR-599
ABCA1	hsa-miR-33b-5p
ABCA1	hsa-miR-425-5p
ABCA1	hsa-miR-758-3p
ABCA1	hsa-miR-454-3p
ABCA1	hsa-miR-199b-3p
ABCA1	hsa-miR-130a-5p
ABCA1	hsa-miR-219a-2-3p
ABCA1	hsa-miR-340-5p
ABCA1	hsa-miR-300
ABCA1	hsa-miR-450b-5p
ABCA1	hsa-miR-873-5p
ABCA1	hsa-miR-543
ABCA1	hsa-miR-374b-5p
ABCA1	hsa-miR-760
ABCA1	hsa-miR-301b-3p
ABCA1	hsa-miR-1294
ABCA1	hsa-miR-1297
ABCA1	hsa-miR-302e
ABCA1	hsa-miR-4295
ABCA1	hsa-miR-3666
ABCA1	hsa-miR-5195-3p
ABCA1	hsa-miR-5590-3p
IL6R	hsa-let-7a-5p
IL6R	hsa-let-7b-5p
IL6R	hsa-let-7c-5p
IL6R	hsa-let-7e-5p
IL6R	hsa-let-7f-5p
IL6R	hsa-miR-21-5p
IL6R	hsa-miR-22-3p

mRNA	miRNA
IL6R	hsa-miR-23a-3p
IL6R	hsa-miR-98-5p
IL6R	hsa-miR-34a-5p
IL6R	hsa-miR-212-3p
IL6R	hsa-let-7g-5p
IL6R	hsa-let-7i-5p
IL6R	hsa-miR-23b-3p
IL6R	hsa-miR-124-3p
IL6R	hsa-miR-125b-5p
IL6R	hsa-miR-138-5p
IL6R	hsa-miR-125a-5p
IL6R	hsa-miR-320a
IL6R	hsa-miR-34c-5p
IL6R	hsa-miR-330-3p
IL6R	hsa-miR-449a
IL6R	hsa-miR-451a
IL6R	hsa-miR-491-5p
IL6R	hsa-miR-495-3p
IL6R	hsa-miR-496
IL6R	hsa-miR-515-5p
IL6R	hsa-miR-506-3p
IL6R	hsa-miR-590-5p
IL6R	hsa-miR-449b-5p
IL6R	hsa-miR-130a-5p
IL6R	hsa-miR-320b
IL6R	hsa-miR-320c
IL6R	hsa-miR-1321
IL6R	hsa-miR-320d
IL6R	hsa-miR-4319
IL6R	hsa-miR-23c
IL6R	hsa-miR-4458
IL6R	hsa-miR-4500
IL6R	hsa-miR-5688
PLAUR	hsa-miR-193a-3p
PLAUR	hsa-miR-377-3p
PLAUR	hsa-miR-335-5p
PLAUR	hsa-miR-193b-3p
PLAUR	hsa-miR-340-5p

Table S5. Results of GO and KEGG Enrichment Analysis for hub genes

ONTOLOGY	ID	Description	GeneRatio	BgRatio	pvalue	p.adjust	qvalue
BP	GO:0048661	positive regulation of smooth muscle cell proliferation	3/6	98/18870	2.69E-06	6.55E-04	1.75E-04
BP	GO:0032755	positive regulation of interleukin-6 production	3/6	101/18870	2.94E-06	6.55E-04	1.75E-04
BP	GO:0032642	regulation of chemokine production	3/6	104/18870	3.21E-06	6.55E-04	1.75E-04
BP	GO:0032602	chemokine production	3/6	105/18870	3.31E-06	6.55E-04	1.75E-04
BP	GO:0002526	acute inflammatory response	3/6	107/18870	3.50E-06	6.55E-04	1.75E-04
CC	GO:0009897	external side of plasma membrane	4/6	387/19886	2.05E-06	7.60E-05	3.89E-05
CC	GO:0045335	phagocytic vesicle	2/6	142/19886	7.45E-04	1.17E-02	5.97E-03
CC	GO:0042581	specific granule	2/6	160/19886	9.45E-04	1.17E-02	5.97E-03
CC	GO:0045121	membrane raft	2/6	286/19886	2.98E-03	2.02E-02	1.04E-02
CC	GO:0098857	membrane microdomain	2/6	287/19886	3.00E-03	2.02E-02	1.04E-02
MF	GO:0002020	protease binding	2/6	142/18496	8.60E-04	2.56E-02	8.50E-03
MF	GO:0019955	cytokine binding	2/6	145/18496	8.97E-04	2.56E-02	8.50E-03
MF	GO:0005126	cytokine receptor binding	2/6	273/18496	3.13E-03	3.27E-02	1.09E-02
MF	GO:0008035	high-density lipoprotein particle binding	1/6	13/18496	4.21E-03	3.27E-02	1.09E-02
MF	GO:0004707	MAP kinase activity	1/6	16/18496	5.18E-03	3.27E-02	1.09E-02
KEGG	hsa04932	Non-alcoholic fatty liver disease	3/6	157/8875	1.04E-04	7.46E-03	3.47E-03
KEGG	hsa05205	Proteoglycans in cancer	3/6	204/8875	2.27E-04	7.46E-03	3.47E-03
KEGG	hsa0541	Lipid and	3/6	216/88	2.69E-	7.46E-	3.47E-

ONTOLOGY	ID	Description	GeneRatio	BgRatio	pvalue	p.adjust	qvalue
	7	atherosclerosis		75	04	03	03
KEGG	hsa05163	Human cytomegalovirus infection	3/6	226/8875	3.08E-04	7.46E-03	3.47E-03
KEGG	hsa05171	Coronavirus disease - COVID-19	3/6	238/8875	3.59E-04	7.46E-03	3.47E-03

GO，Gene Ontology；KEGG，Kyoto Encyclopedia of Genes and Genomes；BP，Biological Process；CC，Cellular Component；MF，Molecular Function。

Table S6. Results of GSEA between LDH and Control group

Description	setSize	enrichmentScore	NES	pvalue	p.adjust	qvalue
HOLLERN_EMT_BREAST_TUMOR_UP	135	0.383860992	1.595757709	1.01E-03	1.02E-02	7.71E-03
WU_APOPTOSIS_BY_CDKN1A_VIA_TP53	51	-0.602959179	-2.178560469	1.36E-06	3.21E-05	2.43E-05
CROONQUIST_IL6_DEPRIVATION_DN	92	-0.559262123	-2.265616437	2.72E-08	9.09E-07	6.88E-07
MANALO_HYPOXIA_DN	275	-0.570051057	-2.650221758	1.00E-10	5.13E-09	3.89E-09

GSEA，Gene Set Enrichment Analysis，LDH，Lumbar Disc Herniation。

Table S7. List of the suggested drugs for LDH

Term	Adjusted P-value	Combined Score	Genes
thalidomide CTD 00006858	0.00004663	4975.730017	ABCA1;PLAUR;TNF;ELANE
Malondialdehyde CTD 00006237	0.001538581	9904.857801	ABCA1;TNF
1,3-Dimethylthiourea CTD 00001818	0.001538581	8291.819666	TNF;ELANE
naringin BOSS	0.001538581	5475.88964	ABCA1;TNF
cannabidiol BOSS	0.001538581	5475.88964	ABCA1;TNF
fenbendazole CTD 00005958	0.001538581	4896.915145	IL6R;TNF
mebendazole CTD 00006249	0.001538581	4647.62851	IL6R;TNF
nitroglycerin CTD 00006039	0.001538581	4647.62851	TNF;ELANE
cytochalasin D BOSS	0.001538581	4647.62851	ABCA1;TNF
Antimycin A CTD 00005427	0.001538581	4420.50837	IL6R;TNF
